# Developing an Intelligent Automatic Appendix Extraction Method from Ultrasonography Based on Fuzzy ART and Image Processing

**DOI:** 10.1155/2015/389057

**Published:** 2015-05-18

**Authors:** Kwang Baek Kim, Hyun Jun Park, Doo Heon Song, Sang-suk Han

**Affiliations:** ^1^Department of Computer Engineering, Silla University, Busan 617-736, Republic of Korea; ^2^Department of Computer Engineering, Pusan National University, Busan 609-735, Republic of Korea; ^3^Department of Computer Games, Yong-In Songdam College, Yongin 449-040, Republic of Korea; ^4^Department of Radiology, Inje University Busan Paik Hospital, Busan 614-735, Republic of Korea

## Abstract

Ultrasound examination (US) does a key role in the diagnosis and management of the patients with clinically suspected appendicitis which is the most common abdominal surgical emergency. Among the various sonographic findings of appendicitis, outer diameter of the appendix is most important. Therefore, clear delineation of the appendix on US images is essential. In this paper, we propose a new intelligent method to extract appendix automatically from abdominal sonographic images as a basic building block of developing such an intelligent tool for medical practitioners. Knowing that the appendix is located at the lower organ area below the bottom fascia line, we conduct a series of image processing techniques to find the fascia line correctly. And then we apply fuzzy ART learning algorithm to the organ area in order to extract appendix accurately. The experiment verifies that the proposed method is highly accurate (successful in 38 out of 40 cases) in extracting appendix.

## 1. Introduction

The appendix vermiformis is a vestigial, tubular organ that arises from the inferior pole of the cecum, 2–2.5 cm inferior to the ileocecal junction. Normal appendix varies in length from 5 to 35 cm (average 8 cm) in adult. It is seen as a blind-ended tubular structure whose orifice is usually constant in position; however, its body and tip are located in various sites including retrocecal (behind the cecum), pelvic, subcecal (below the cecum), and pre- or postileal (anterior or posterior to the terminal ileum) [1, 2].

Appendicitis, an inflammation of the appendix, is the most common abdominal surgical emergency. It is believed to occur as a result of appendiceal luminal obstruction which is most commonly caused by a fecalith. Luminal bacteria multiply and invade the appendiceal wall as venous engorgement and subsequent arterial compromise result from the high intraluminal pressures. Finally, gangrene and perforation occur. If the process evolves slowly, adjacent organs such as the terminal ileum, cecum, and omentum may wall off the appendiceal area so that a localized abscess will develop, whereas rapid progression of vascular impairment may cause perforation with free access to the peritoneal cavity.

Typically, the illness begins with vague midabdominal discomfort followed by nausea, anorexia, and indigestion and within several hours the pain migrates to the right lower quadrant. Examination at this point shows localized tenderness to one-finger palpation and perhaps slight muscular guarding. Rebound or percussion tenderness (the latter provides the same information more humanely) may be elicited in the same area [3].

However, there are various kinds of difficulties in the diagnosis of acute appendicitis. The classic sequence of symptoms occurs in only 66% of patients. And the diagnosis of acute appendicitis is particularly difficult in the very young and in the elderly. These are the groups in which diagnosis is most often delayed and perforation most common due to the lack of classic symptoms. The highest incidence of false-positive diagnosis (20%) is in women between ages 20 and 40 and is attributable to pelvic inflammatory disease and other gynecologic conditions [4]. Diagnosing appendicitis in pregnancy also can be difficult because the nausea, vomiting, and abdominal pain of appendicitis can also be features of pregnancy and physical examination may not be reliable in them [5].

As a result, imaging diagnosis such as ultrasonography (US), computed tomography (CT), and magnetic resonance imaging (MRI) is essential in confirming or excluding the diagnosis of acute appendicitis in clinically suspected acute appendicitis. Among these modalities, US examination does a key role in the management of the clinically suspected appendicitis. US examination should be the first imaging test performed, particularly among the pediatric and young adult populations, who represent the main targets for appendicitis, as well as in pregnant patients. A positive US examination for appendicitis or an alternative diagnosis of possible gastrointestinal or urological origin, or a negative US, either showing a normal appendix or presenting low clinical suspicion of appendicitis, should lead to a final diagnosis. A negative or indeterminate examination with a strong clinical suspicion of appendicitis should be followed by a CT scan or alternatively, a MRI scan in a pregnant patient. A second US examination in a patient with persistent symptoms, especially if the first one was performed by a less-experienced imaging professional, is a valid alternative to a CT [6, 7].

Sonographic findings of acute appendicitis include outer appendiceal diameter enlarged to 6 mm or greater under compression, intraluminal fluid, lack of compressibility, visualization of appendicolith, increased color signals along its wall, cecal wall thickening, periileal lymph nodes, and peritoneal fluid. Among these findings, a threshold 6 mm diameter of the appendix under compression is the most accurate US finding for appendicitis [8]. Thus, the critical point, 6 mm of the diameter of the appendix is a crucial factor in decision making for appendectomy. As a result, the measurement error of 1 mm near the critical point may lead doctors to a serious misdiagnosis.

By the way, current naked-eye examination of the US images has some limitations in accurate measurement in cases of unclear delineation of the appendix with thick abdomen and in cases showing ill-defined borders of the appendix by surrounding tissues. Thus, the need for more clear delineation and accurate extraction of appendix from surrounding tissues is always present to radiologists in the field of abdominal imaging.

Thus, there are growing needs for an intelligent decision tool for more accurate diagnosis by artificial intelligence technology. Unfortunately, there are few tools for the practitioners to use with credibility up to date. A preliminary study applies several histogram thresholding methods in detecting appendix [9] but that method is weak when the brightness contrast is not very high and will have potential information loss in edge linking procedure. Our previous study [10] used fuzzy logic in binarization procedure to enhance the brightness contrast and other studies used *K*-means clustering [11] method to extract the target appendix as accurate as possible. With such a pixel clustering method, the appendix area has more contrast in extraction; however, the correct extraction rate is below satisfaction because patient's ascites of a significant size may mislead the system to extract it as a false positive appendix or when the shape of appendix is extraordinary, the system could not catch the correct fascia line that is the main predictor of appendix location of our logic.

**Figure 1:**
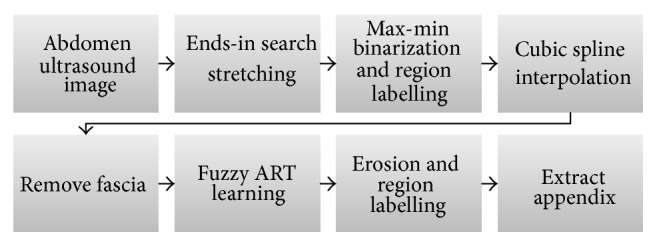
Process of Appendix extraction.

Thus, in this paper, we propose a more efficient method to extract appendix area correctly by using fuzzy ART algorithm in the critical phase instead of *K*-means. Knowing that the appendix is located at the lower organ area below the bottom fascia line, we conduct a series of image processing techniques to find the fascia line correctly.  Figure 1 demonstrates the overall process of our method.

The first step is to enhance the brightness contrast by Ends-in Search Stretching [12] and remove noises by Max-Min binarization and region labeling method. Then the fascia area is extracted with cubic spline interpolation [13]. The appendix area is then extracted from that image by applying fuzzy ART algorithm as explained in Section 3.

## 2. Removing Fascia Area

**Figure 2:**
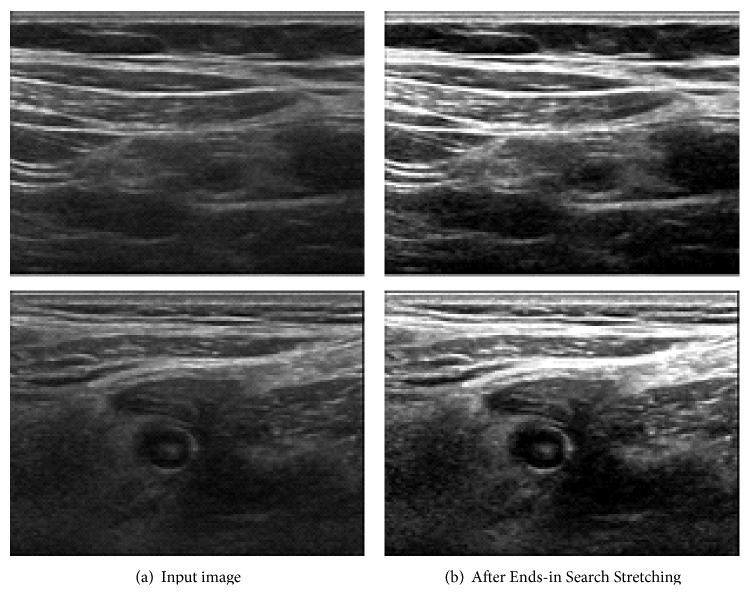
Effect of Ends-in Search Stretching.

First, we apply End-in Search Stretching [12] to enhance the brightness contrast with the formula (1). Since the abdomen image is usually dark, it may not be sufficient to discriminate fascia, muscles, and other areas as it is given. Consider the following:

(1) Sx,y =0Px,y≤Min⁡255×Px,y−Min⁡Max⁡−Min⁡Min⁡<Px,y<Max⁡255Px,y≥Max⁡,

where Min and Max are thresholds, *P*(*x*, *y*) denotes the brightness value in the original image, and *S*(*x*, *y*) denotes the result.  Figure 2 shows the effect of Ends-in Search Stretching. Two different input images shown in Figure 2(a) become more brightness contrast as shown in Figure 2(b). Throughout the paper, we will show two example images at the same time in this section so that the effect of our image processing subsystems can be identified more clearly.

**Figure 3:**
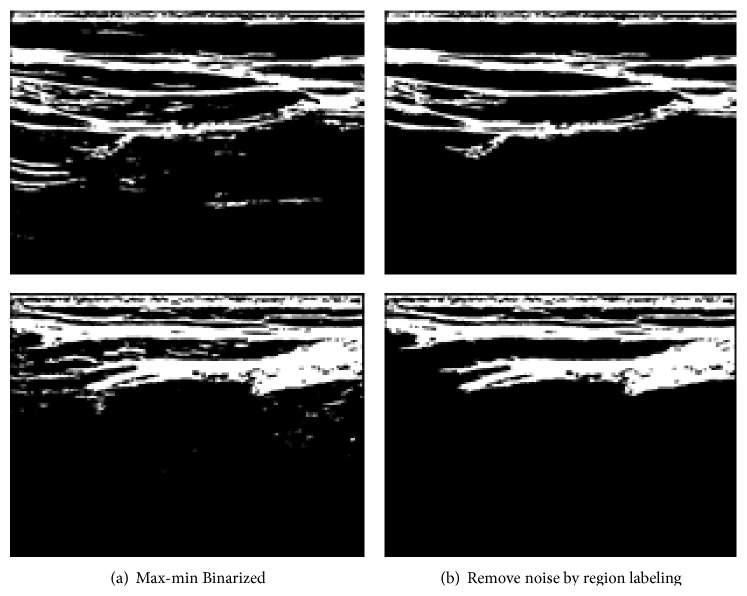
Effect of Region Labeling.

From Figure 2(b), we apply Max-Min binarization and repetitive region labeling method [14] to connect related pixels. If the connected object is too short, we remove them as noise. The result is as shown in Figure 3(b) and the experimental threshold in this paper is 1500.

Unfortunately, the binarized noise-removed image may have disconnected fascia area at the bottom due to the brightness difference of that area. In order to reconnect them, we apply cubic spline interpolation [13].

Cubic spline interpolation connects two points on the boundary when formula (2) is satisfied:

(2) $$\begin{array}{c} S_{n-1}^{\prime \prime }\left(x_{n}\right)=S_{0}^{\prime \prime }\left(x_{0}\right)=0\\ S_{i-1}^{\prime \prime }\left(x_{i}\right)=S_{i}^{\prime \prime }\left(x_{i}\right)\;i=1,2,\ldots ,n-1\\ S_{i-1}^{\prime }\left(x_{i}\right)=S_{i}^{\prime }\left(x_{i}\right)\;i=1,2,\ldots ,n-1\\ S_{i-1}^{}\left(x_{i}\right)=S_{i}^{}\left(x_{i}\right)=y_{i}\;i=1,2,\ldots ,n-1. \end{array}$$

**Figure 4:**
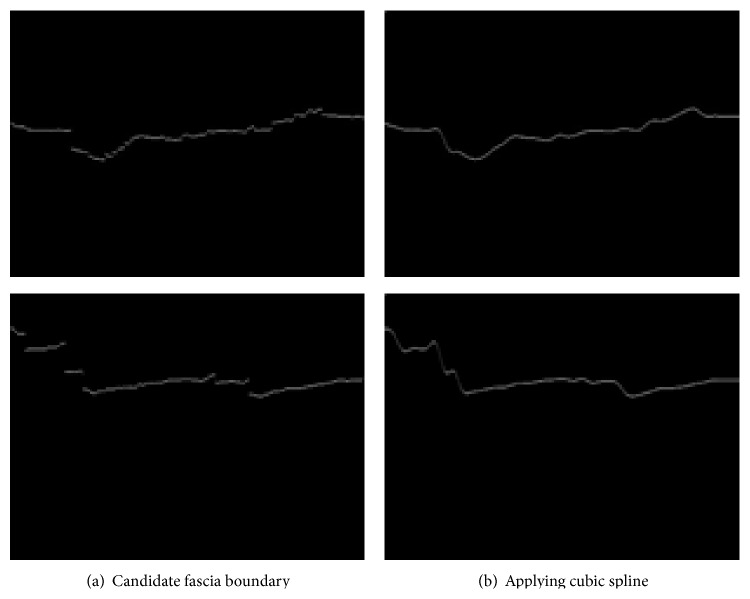
The effect of cubic spline interpolation.

First two equations are hard constraints but the next two are soft constraints for smoothing. Since we know that the target appendix is located below the bottom of fascia, cubic spline interpolation is applied to the bottom fascia line and the effect is shown in Figure 4.

## 3. Extract Appendix Object from Image

In order to extract the candidate area of appendix, we apply an unsupervised neural network learning algorithm called fuzzy ART [15] to the image obtained from Section 2.

The general characteristics of fuzzy ART can be summarized as follows:

1. an unsupervised real time learning algorithm that does not have the target value,
2. it creates a new cluster or merges existing clusters according to the similarity between input pattern and current set of clusters.

The main reason we adopt an unsupervised learning algorithm for our system is that the supervised learning suffers from frequent relearning of the learned patterns. Fuzzy ART algorithm is relatively immune to that problem since it has incremental learning capability and is also proven to be stable in learning [16].

**Figure 5:**
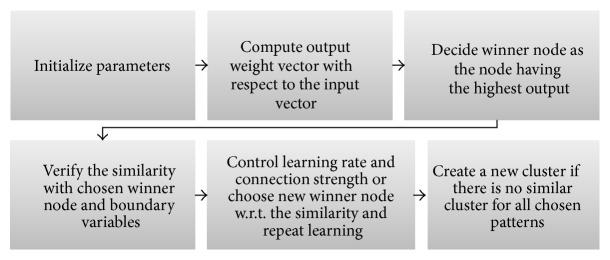
Process diagram for fuzzy ART.

The process of fuzzy ART learning in this problem can be summarized as in Figure 5.

The similarity of the input patterns is computed as formula (3) where ∧ denotes fuzzy logic Min operator:

(3) $$\begin{array}{c} \frac{\left\|w_{j^{*}i}\left(n\right)\wedge x_{i}\right\|}{\left\|\beta_{\max}\right\|}>\rho , \end{array}$$

where *x*_*i*_ denotes the normalized brightness value of current pixel within [0,1] and *β*_max⁡_ denotes the normalized maximum brightness value. And *w*_*j*^∗^*i*_(*n*) denotes the weight of connection between all *i* nodes in the input layer and the node*j* chosen as a winner node in the cluster layer and *ρ* denotes the vigilance parameter.

The output (*O*_*j*_) is computed as formula (4) and the winner node is determined as having the maximum output as shown in formula (5):

(4) $$\begin{array}{c} O_{j}=\frac{\left\|w_{ji}\left(n\right)-x_{i}\right\|}{\left\|\beta_{\max}\right\|}, \end{array}$$

(5) $$\begin{array}{c} O_{j^{*}}=\vee \left(O_{j}\right). \end{array}$$

Then the weight *w*_*j*^∗^*i*_(*n* + 1) is controlled as formula (6):

(6) $$\begin{array}{c} w_{j^{*}i}\left(n+1\right)=\alpha \left(x\wedge w_{j^{*}i}\left(n\right)\right)+\left(1-\alpha \right)w_{j^{*}i}\left(n-1\right), \end{array}$$

where the learning parameter *α* is a real value between 0 and 1.

**Figure 6:**
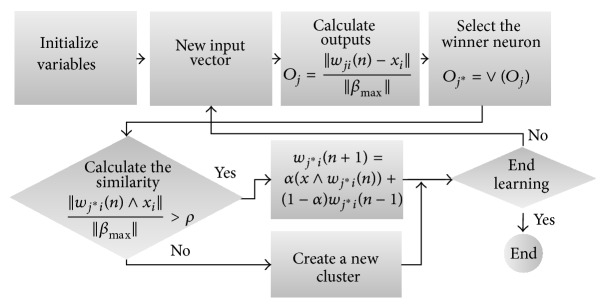
Distance based fuzzy ART process flow diagram.

Then, the detailed algorithmic description of fuzzy ART process flow diagram is shown in Figure 6.

The main reason that we adopt fuzzy ART in extracting appendix area is to avoid cases when patient's ascites with a significant size are falsely classified as appendix. In such cases, the brightness difference itself is not sufficient to discriminate those two objects differently. That is one of the limitations of the previous research [11] using *K*-means pixel clustering. That problem is caused by the nature of *K*-means in that the clusters are based on random brightness values as centers.

The characteristics of fuzzy ART, meanwhile, is to determine single winner node with respect to the minimum error between input and weighted pattern based on Euclidean distance metric. Thus, it is possible to discriminate ascites from appendix in brightness by checking boundary variables in fuzzy ART algorithm.

Knowing that the appendix has the shape of oval with low brightness, we apply erosion operation and region labeling method to extract the right area for the appendix with noise removal.

## 4. Experiment and Analysis

**Figure 7:**
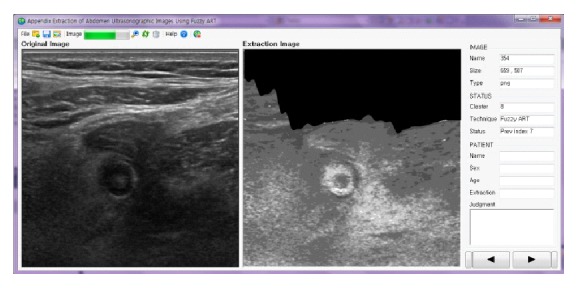
Screenshot of proposed software.

**Figure 8:**
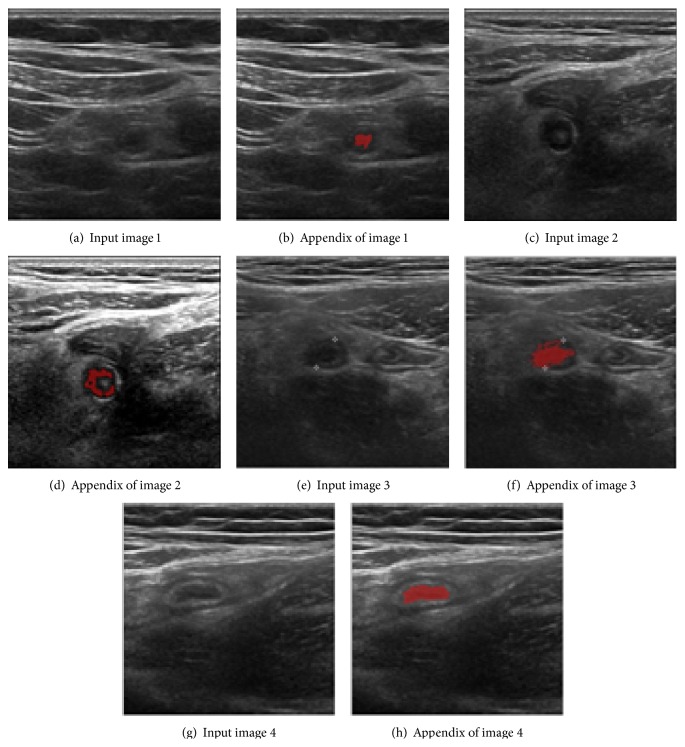
Examples of experiment result.

The system is implemented in Visual Studio 2010 C# with Intel(R) Core(TM) i7-2600 CPU @ 3.40 GHz and 4 GB RAM PC. Forty images containing appendicitis supplied by Busan Paik Hospital and Busan National University Medical Center are used in this experiment. The actual system gives some characteristic features of extracted appendix as shown in Figure 7. Four different example appendicitis cases extracted by our proposed method are demonstrated in Figure 8.

**Figure 9:**
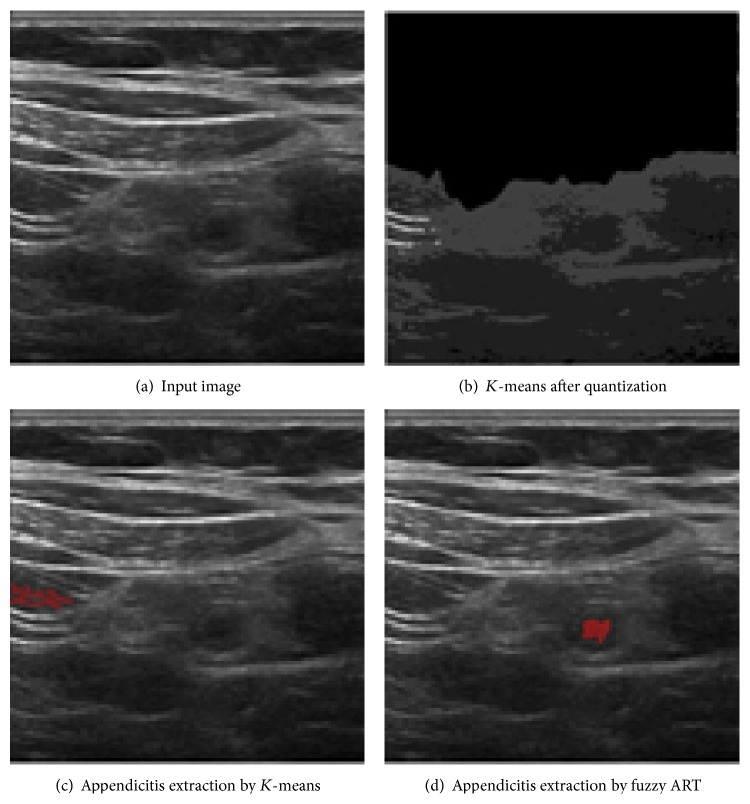
False positive case of *K*-means compared with proposed fuzzy ART in extraction.

In Figure 9, we provide a visual comparison between the proposed method and the reimplemented version of previous *K*-means clustering based method [10]. By the nature of the algorithm, *K*-means pixel clustering is sensitive to the initial brightness values. From the same input image shown in Figure 9(a), *K*-means tends to cause clustering errors as shown in Figure 9(c) when there exists sufficiently large noise not removed in the quantization process shown in Figure 9(b).

Our proposed method based on the fuzzy ART algorithm is, however, relatively immune to that brightness sensitivity and extract appendix effectively as shown in Figure 9(d) from the same input shown in Figure 9(a). In fuzzy ART process, even though the brightness contrast between the center and the observed pixel is small, the proposed system takes into account the current information with its previous state in weight control process so that clustering errors are minimized.

**Figure 10:**
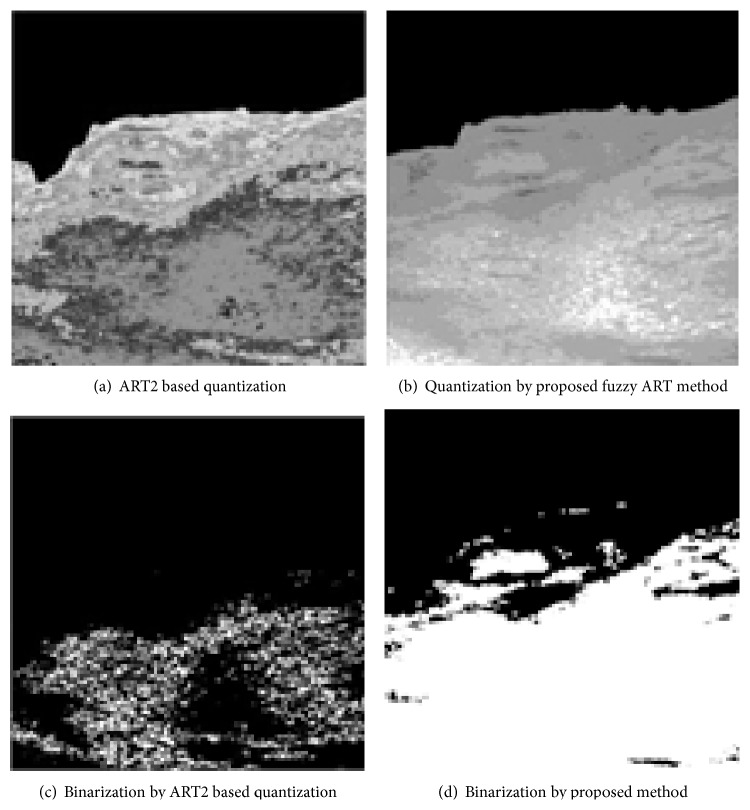
Quantization and binarization by ART2 and by proposed method.

Also, the fuzziness of the proposed method is crucial in appendicitis extraction. Even within the same ART family, if we use ART2 algorithm [17] without fuzzy control, the performance is less than satisfactory.  Figure 10 demonstrates the comparison of quantization and binarization by ART2 and proposed fuzzy ART.

Quantization result by ART2 shown in Figure 10(a) shows failed candidate area extraction of the target appendix. That is due to the sensitivity of vigilance parameter setting by the nature of ART2. In the clustering process, ART2 tends to have too many clusters than desired with respect to the setting of vigilance parameters; thus, similar characteristics of the appendix may belong to different clusters. Specifically, for the example shown in Figure 10, ART2 had 14 clusters whereas our proposed method had 8 clusters and resulted in much better result as shown in Figure 10(b). That is because fuzzy ART is relatively not sensitive to the setting of vigilance parameter; thus, our method shows more stable performance. It is worse when the binarization process is performed after such quantization. As one can see in Figure 10(c), the binarization process based on the incorrect ART2 clustering of Figure 10(a) results in the black area that the appendix and the background are inseparable whereas our proposed method has little difficulty to extract the appendix area correctly as shown in Figure 10(d). Thus we may conclude that the fuzzy control of clustering process is essential for the performance stability.

In conjunction with the false positive problem caused by ascites detected from previous study [10], the proposed system shows much better extraction rate as shown in Table 1. The success and failure decision of the automatic extraction by the system is determined by field expert.

Since all 40 images used in the experiment contain appendicitis, there is no true negative case and all three methods conclude an extraction of appendicitis; thus, there is no false negative either. The sensitivity of the appendicitis extraction is greatly improved from 67.5% (*K*-means based) and 82.5% (ART2 based) to 95% by the proposed method.

From the related literature, our result is much better than previous appendix segmentation from ultrasound image by histogram thresholding which was greatly sensitive to the probing position [9] and the extraction accuracy is very close to that of CT result reported (33 out of 34 cases) [5].

**Figure 11:**
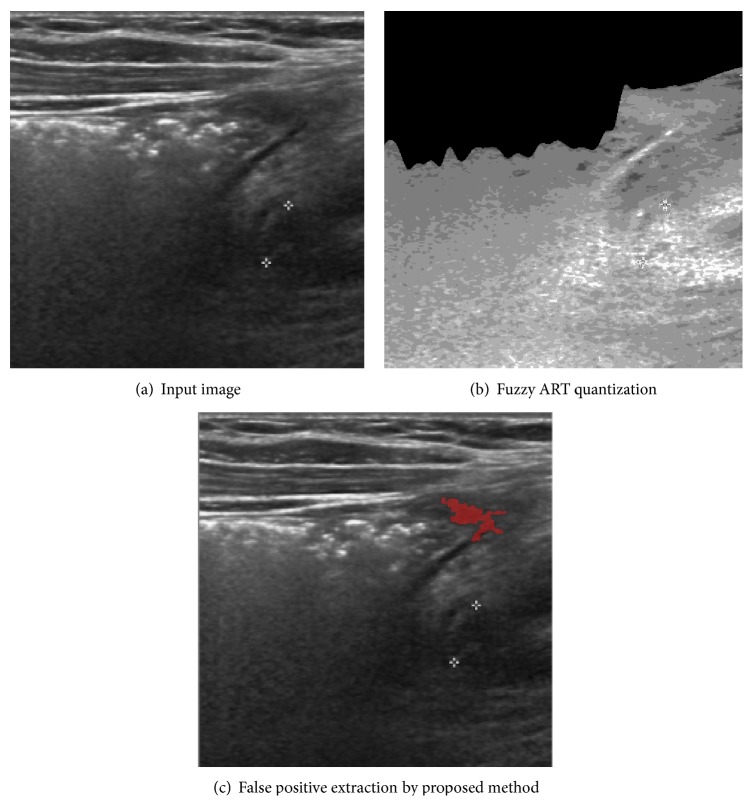
Failed appendicitis extraction case by proposed method.

Unfortunately, there are two failed extraction cases by our proposed method as well. As one can see from Figure 11(a), there is almost no distinction between the appendicitis area and its neighbor background. Although our proposed method applies careful brightness contrast enhancement procedures, the quantized result as shown in Figure 11(b) gives little clue in clustering and as a result, such clustering errors cause false extraction of appendicitis. In order to overcome this huddle, we need other morphological attributes in consideration such as the elliptical shape of the appendix of a certain size in the future research.

## 5. Conclusion

In this paper, we propose a method to extract appendicitis from ultrasound image automatically with various image processing techniques and fuzzy ART learning algorithm.

Knowing that the appendix is below fascia area; thus, we try to find fascia area first and remove it from our region of interest. In this part, the bottom fascia lines were carefully treated with cubic spline interpolation such that the lines were connected correctly. Many other image processing techniques such as Ends-in Search Stretching, Max-Min binarization, and region growing labeling are used to enhance the brightness contrast, remove noises, and connect pixels. Then the fuzzy ART learning is applied to classify pixels into the same objects of their labels such that the appendicitis could be extracted based on its morphological features.

Extracted appendix results were shown to medical experts and the proposed method shows a clear improvement from the previous study [10] in that our method is now successfully discriminate patient's ascites of a significant size case from appendix.

Developing automatic appendicitis diagnosis software to assist medical doctors is the final goal of our research and we believe that the proposed method in this paper can be an important building block of such effort.

## Figures and Tables

**Table 1:** Extraction accuracy comparison (40 cases).

Method	True positive	True negative	False positive	False negative
*K*-means based	27	0	13	0
ART2 based	33	0	7	0
Proposed system	38	0	2	0
